# The influence of human population movement on mass drug administration for neglected tropical diseases: a scoping review

**DOI:** 10.1186/s40249-026-01433-w

**Published:** 2026-04-07

**Authors:** Moussa Sangare, Oumar Coulibaly, Claudia Duguay, Abdoul Fatao Diabate, Dukharmel Nazaire, Kiflom Hailu, Yaya Ibrahim Coulibaly, Carol Vlassoff, Manisha A. Kulkarni, Alison Krentel

**Affiliations:** 1 International Center of Excellence in Research in Mali, University of Sciences, Techniques, and Technologies of Bamako, Bamako, Mali; 2 Interdisciplinary School of Health Sciences, University of Ottawa, Ottawa, ON Canada; 3 School of Epidemiology and Public Heath, Faculty of Medicine, University of Ottawa, Ottawa, ON Canada; 4 Bruyère Health Research Institute, University of Ottawa, Ottawa, ON K1N 5C8 Canada

**Keywords:** Population mobility, Mass drug administration, Neglected tropical diseases, Africa, Coverage, Scoping review

## Abstract

**Background:**

Neglected tropical diseases (NTDs), including but not limited to lymphatic filariasis, onchocerciasis, trachoma, schistosomiasis, and soil-transmitted helminths, remain a major public health challenge in Africa. Mass drug administration (MDA) is a key strategy for NTD control and elimination. However, mobile populations such as internally displaced persons (IDPs), nomadic groups, and seasonal workers often face major constraints that reduce MDA effectiveness defined by coverage. Understanding how mobility and its underlying drivers affect MDA implementation is therefore critical to designing inclusive, effective, and equitable intervention strategies. In this review, we aim to map existing evidence on how human population movement influences the outcomes of MDA programs in Africa.

**Methods:**

Following PRISMA-ScR guidelines, we searched using electronic databases Medline (Ovid), Embase, Web of Science, and manually screened additional sources for studies published in English or French between January 2000 and February 19, 2025. Two authors independently extracted data, resolving discrepancies through discussion, or with a third reviewer. We included studies reporting on MDA interventions and mobile or migrant populations in Africa. Data were extracted using a standardized template and synthesized thematically to describe mobility patterns, barriers to access, implementation gaps, and strategies used to improve MDA reach among mobile groups. The thematic synthesis consisted of organizing the extracted data into recurring themes, comparing trends across studies, and grouping similar observations to develop broader themes that reflect common challenges and approaches related to mobile populations.

**Results:**

From 6814 studies identified, twenty (20) met the inclusion criteria. The review identified multiple challenges likely to affect MDA equity and effectiveness. Mobility, particularly among nomad pastoralists, seasonal workers, IDPs, and cross-border populations leads to systematic exclusion from MDA campaigns. Barriers included geographic inaccessibility, limited tailored communication, lack of cross-border collaboration/coordination, and rigid campaign schedules. Promising strategies documented were mobility-informed microplanning, cross-border collaboration/coordination, flexible delivery models, enhanced community engagement and integration with other health interventions. The evidence emphasizes the need for adaptive, equity-focused MDA approaches to effectively reach mobile populations.

**Conclusions:**

Population mobility has a significant impact on equitable MDA delivery and can hinder progress toward NTD elimination targets in Africa. Tailored, flexible, and inclusive strategies are urgently needed to ensure mobile populations are reached. Future efforts should focus on developing mobility indicators, pilot-testing adaptive MDA delivery models aligned with movement patterns and strengthening partnerships with neighboring countries and humanitarian organizations.

**Supplementary Information:**

The online version contains supplementary material available at 10.1186/s40249-026-01433-w.

## Background

Neglected tropical diseases (NTDs) comprise a group of 21 diseases and conditions that disproportionately affect people living in vulnerable circumstances, particularly in underserved and hard-to-reach areas [1]. Among these, five diseases including lymphatic filariasis, onchocerciasis, schistosomiasis, soil-transmitted helminthiases, and trachoma are classified as NTDs that can be addressed by the use of preventive chemotherapy (PC-NTDs) because they can be controlled or eliminated through large-scale mass drug administration (MDA). The African region carries a disproportionately high burden of these PC-NTDs, with the majority of global cases occurring on the continent [1, 2]. Overall, Africa bears 39% of the total global NTD burden. However, PC-NTDs are preventable, treatable, and through sustained interventions, have the potential to be eliminated. Global efforts against NTDs have seen significant progress. The number of people globally needing interventions for NTDs fell by 32% from 2010 to 2023, and by May 2025 a major milestone was reached with 56 nations worldwide having eliminated at least one NTD [3]. This progress aligns with WHO's global target of achieving elimination of at least one NTD in 100 countries by 2030 [3]. These achievements highlight the feasibility of NTD elimination when appropriate strategies are implemented effectively and are sustained.

Mass drug administration (MDA) has been a cornerstone of global and regional strategies to eliminate the PC-NTDs as public health problems. MDA is a safe and inexpensive means of delivery of essential drugs based on the principles of preventive chemotherapy, whereby populations or sub-populations are offered treatment regardless of their disease status [4]. However, despite the scale-up of MDA programs across the continent, persistent coverage gaps remain, especially among mobile and migrant populations (MMPs) who often temporarily reside or move across endemic regions [5–7]. There is growing evidence that population growth and mobility are impeding PC-NTDs elimination [8]. In the context of this paper, ‘mobile populations’ refer to groups with limited fixed residence whose movements are driven by livelihood, socio-economic, or security factors. Examples include nomadic pastoralists, internally displaced persons (IDPs), seasonal agricultural and mining workers, refugees, seasonal migrants, and those engaged in cross-border movements. This raises the possibility that NTDs could re-establish in areas where they have been eliminated. For example, NTD-affected mobile populations, such as nomadic pastoralist communities, IDPs, seasonal workers (e.g. agricultural, mining), refugees, migrants and cross-border movement groups, may migrate to areas where MDA has stopped, based on achieving target prevalence or where disease prevalence is so low that transmission is no longer a concern.

NTD initiatives often face challenges to reach mobile populations. These population groups have limited geographic access to general health services including NTDs, and may experience poor quality of care, interruptions in treatment delivery, and high costs associated with travel and care-seeking [9] These challenges are compounded by linguistic barriers, low awareness of NTDs, and alternative health beliefs, which hinder uptake of prevention and treatment strategies [10–12]. Barriers also include geographic inaccessibility, population movement and related professions, sociopolitical marginalization, conflict and insecurity, limited integration in national health systems, and weak surveillance and follow-up mechanisms. Cultural norms, language diversity, and irregular documentation status can further complicate service provision. [5, 7, 13]. These barriers to prevention and treatment may contribute to transmission hotspots, putting hard won progress at risk and potentially leading to disease re-emergence [14]. These barriers highlight a critical need to better tailor MDA approaches to populations on the move.

While global NTD strategies recognize the importance of equity and inclusivity in disease elimination, there remains a limited understanding of how population mobility impacts MDA delivery, uptake, and effectiveness in African contexts. Existing research is fragmented and often focuses on fixed communities, overlooking the complex realities of population movement and health service delivery at the margins. Mobility involving IDPs, nomadic pastoralists, refugees, migrants, among others is caused by insecurity, climate change and worsened poverty. It has been demonstrated that, in comparison to the general population, these people have the least access to MDA [7]. In the African context, where mobile groups are frequently under -represented in standard health data systems, this gap in the research highlights the urgent need to thoroughly map the nature, magnitude, and impact of population movement on NTD interventions of MDA.

We conducted a scoping review to synthesize the available evidence of population mobility effect on the implementation of MDA for PC-NTDs in Africa. This review aimed to explore the factors driving population movement and to assess their consequences on health outcomes, with a particular focus on the implementation of MDA programs in Africa. The overall goal was to inform more inclusive, equity-driven strategies for NTDs elimination that are responsive to the needs of mobile and migrant populations. Specifically, this review sought to address the following questions: Why are these populations moving? Where are these populations coming from or going to? What challenges are associated with the ability and willingness of stakeholders, including the beneficiary populations, to successfully implement MDA to effectively control/eliminate NTDs and other infectious diseases?

### Operational definitions of concepts

#### Mobile populations

We defined population mobility as the geographic movement of people from their usual place of residence to another location for reasons that include seeking better economic or agricultural opportunities, escaping natural disasters or conflict, or finding safer living conditions. It may be from one country to another, from one region to another, or from rural to metropolitan areas, depending on the reason for the relocation. Nomadic pastoralists, livestock breeders, refugees, IDPs, and migrants for informal economic purposes are examples of mobile populations. They frequently lack uninterrupted access to basic services like healthcare, education, and social security, making them vulnerable [15, 16]. IDPs, refugees, informal economic migrants, and nomadic pastoralists, especially livestock producers, were the MMP included in the focus for this study.

#### Mass drug administration (MDA)

Is a campaign approach that provides preventive chemotherapy to all eligible individuals in a disease endemic region, irrespective of their infection status. The WHO advises MDA on a periodic basis according to the community's infection level. Reaching the WHO-recommended threshold coverage levels is essential to control and eliminate NTDs: (1) LF: > 65% of the entire population at risk, focusing on all eligible individuals; (2) schistosomiasis: > 75% of school-age children (and at-risk adults in certain situations); (3) onchocerciasis: ≥ 80% of the eligible population (typically people aged 5 and older in endemic areas); (4) soil-transmitted helminthiases (STH): ≥ 75% of preschool- and school-age children; (5) trachoma: ≥ 80% of the population is required in endemic areas (as part of the SAFE (Surgery for trichiasis, Antibiotics to treat infection, Facial cleanliness, and Environmental improvement) strategy, emphasizing on MDA with azithromycin) [17, 18].

## Methods

### Study design

This scoping review followed a previously published protocol [19] in accordance with the Joanna Briggs Institute methodology (JBI) guidance for scoping reviews [20] and the Preferred Reporting Items for Systematic Review and Meta-Analysis Protocols (PRISMA-P) for writing the protocol [21]. To guide the reporting of our scoping review, we used the PRISMA Extension for Scoping Reviews (PRISMA-ScR) (Supplementary file 1. PRISMA-ScR Checklist) [22].

### Eligibility criteria

We used the PCC (Population, Concept, Context) framework [23] to guide the research question and search strategy: (1) Population: We included studies involving mobile and migrant populations in Africa, including nomadic groups, seasonal workers, refugees, IDPs, cross-border migrants; (2) Concept: We focused on studies reporting data on MDA coverage, treatment compliance, transmission indicators, progress toward elimination targets, barriers to MDA access, or strategies to improve coverage and adherence among mobile populations; (3) Context: We limited our review to studies reporting specifically the five PC-NTD diseases (lymphatic filariasis, onchocerciasis, schistosomiasis, soil-transmitted helminthiases, and trachoma). Only studies conducted in African countries endemic for at least one of the targeted diseases were included.

### Inclusion and exclusion criteria

#### Inclusion criteria

We included studies published between January 2000 and February 2025 in English or French. This period corresponds with intensified global NTD elimination efforts (e.g., WHO’s Global Programme to Eliminate Lymphatic Filariasis launched in 2000) [24], improved monitoring tools such as geographic information system and molecular xenomonitoring [25, 26], and increasing population movements driven by migration and humanitarian crises [27].

#### Exclusion criteria

Studies were excluded if they were literature reviews, conference abstracts, protocols, editorials, or commentaries; did not directly focus on mobile or migrant populations and MDA or PC-NTDs; or lacked primary data relevant to the review objectives.

We recognize that these restrictions may constitute limitations of our review. A summary of all eligibility, inclusion, and exclusion criteria is provided in Table 1.

**Table 1:** Inclusion, exclusion, and data extraction criteria

Key themes	Screening phase 1 (title/abstract)	Screening phase 2 (full-text)	Extraction (data to collect)
Mass drug administration (MDA)	Studies mentioning MDA for neglected tropical diseases in any population	Studies explicitly focusing on MDA implementation, challenges, or outcomes in mobile populations	• Target diseases: PC-NTDs (onchocerciasis, lymphatic filariasis, schistosomiasis, soil-transmitted helminthiases and trachoma) • MDA strategy (e.g., fixed-point, mobile teams) • Coverage metrics (e.g., %, denominators)
Mobile population	Studies involving migrants, refugees, nomads, or temporally displaced groups (e.g., seasonal workers)	Detailed analysis of mobility’s impact on MDA access, coverage, or adherence	• Population type (e.g., seasonal workers and migrants, Internally displaced people, nomads and transhumants) • Mobility patterns (cyclical, one-way)
Reasons for movement	Any mention of migration drivers (e.g., conflict, livelihoods, environmental factors)	Clear linkage between movement patterns and MDA delivery (e.g., logistical barriers, coverage gaps)	• Primary drivers (economic, conflict, environmental) • Impact on MDA participation
Consequences on MDA	Studies discussing health program challenges (e.g., low coverage, inequities) in mobile groups	Evidence of how mobility affects MDA success (e.g., • Coverage gaps • Logistical issues • Disease resurgence)	• Quantified gaps (e.g., 30% missed doses) • Program adaptations • Logistical issues • Disease resurgence
Successful approaches	Interventions or strategies (e.g., community engagement, mobile clinics) to improve MDA in mobile populations	Evidence of effectiveness: • Community engagement • Cross-border coordination	• Strategy details • Outcome metrics (e.g., • coverage improvement) Documented outcomes (e.g., increased coverage, reduced prevalence)
Study type	–	Primary research/program evaluations 1. Primary research, evaluations, reports 2. Excludes lab-based or animal studies	• Study design (e.g., cohort, randomized controlled trial) • Data collection methods • Exlude studies NOT relevant to mobile population or MDA
Language	–	Published in English or French	–
Year	Published from 2000–2024	–	Publication year

*PC-NTDs* *Preventive Chemotherapy for Neglected Tropical Diseases* NB: The primary research for this review included systematic reviews, modeling studies, and program evaluations that provided original insights into the research question

### Search strategy and information sources

#### Database searches

Under the guidance of a research librarian of Health Sciences from the University of Ottawa, the study team (MS, OC, CD and AK) devised a comprehensive search strategy using subject headings and keywords was developed to search for eligible published studies in Medline (Ovid), Embase, and Web of Science databases. The searches were constructed by combining search terms as indicated in the Supplementary file 2 (Supplementary file 2. Database final strategy search). Search terms included mobile, nomadic populations, migrant, internally displaced person, access to healthcare, neglected tropical diseases, NTDs, Africa, mass drug administration, MDA, care, movement, strategy and infectious agent and vector of NTDs. Search concepts were combined with “AND”, and words within a word group or with a concept were combined with “OR”. Practical screening assessed the title of the publication as well as date of publication. Using pertinent search terms, the search strategy was restricted to the five WHO preventive chemotherapy NTDs of interest: (1) lymphatic filariasis (or elephantiasis or filarial or *Wuchereria bancrofti* or *Brugia malayi* or *Brugia timori* or microfilaria AND); (2) onchocerciasis (or *Onchocerca volvulus* or river blindness or onchocerciasis or *volvulus* AND); (3) schistosomiasis (or *Schistosoma haematobium* or *Schistosoma intercalatum* or *Schistosoma guineensis* or *Schistosoma mansoni* or *bilharzia* or snail fever AND); (4) soil-transmitted helminthiasis (or *Ascaris lumbricoides* or *Trichuris trichiura* or *Ancylostoma* or *Necator americanus* or roundworm or whipworm or hookworm AND); (5) trachoma (or *Chlamydia trachomatis* or trachoma or granular conjunctivitis AND). Supplementary file 2. Although *Wuchereria bancrofti* is the only causative agent of lymphatic filariasis in Africa, we included *Brugia malayi* and *Brugia timori* in the initial search strategy to ensure sensitivity and comprehensiveness. African studies may reference *Brugia spp*. in broader discussions of lymphatic filariasis or in reviews comparing global species distribution.

#### Hand searches

Additional searches were conducted on the WHO, World Bank websites and the Centers for Disease Control and Prevention (CDC), The International Organization for Migration (IOM), The International Labour Organization (ILO) and the United Nations High Commissioner for Refugees (UNHCR) for unpublished studies. The manual search included the same terms as the database search. We also conducted a hand search of gray literature by reviewing references identified during manual searches and other external sources / websites, although no formal gray literature search strategy or predefined repository was used.

#### Study selection and screening process

We imported all the references/citations we found as well as inclusion/exclusion criteria into Covidence, an online tool that helps manage systematic reviews (https://www.covidence.org/). The platform automatically flagged and removed any duplicate studies. From there, the studies selection process was done in two phases:

### Title and abstract screening

Two independent reviewers screened each study’s title and abstract against our pre-set criteria, guided by the PCC framework [23]. If a study did not focus on mobile populations, MDA, or was not conducted in Africa, we excluded it. Whenever the reviewers disagreed, they discussed until they reached a consensus. All other conflicts between the two reviewers were resolved by a third reviewer.

### Full-text review

For studies that passed the initial screening (Title and abstract), we obtained and carefully read the full articles to confirm whether they fit our criteria. Reasons for exclusion at this stage were documented, and the remaining studies were retained for data extraction.

We documented each step of the process and summarized it in a PRISMA-ScR flow diagram, showing how many records, we started with, how many were screened, included, or excluded along with the reasons for exclusion when applicable. This process ensures that only the most relevant studies were included in our final selection.

### Data charting, collection and extraction procedures

Two independent reviewers extracted the data using a Microsoft Excel spreadsheet (Supplementary file 3. Data extraction sheet.xlsx). adapted from the Joanna Briggs Institute’s data extraction template. The template was piloted on three studies and refined before full application to ensure consistency and relevance. The piloting process was done independently by 2 reviewers followed by a discussion to agree on the final template. The extracted information included: (i) Study characteristics such as the first author's name, year of publication, country of study, and study design; (ii) Intervention details including MDA implementation strategies, facilitators and barriers to delivery, and reported outcomes or impacts; (iii) Population mobility or reasons for movement, patterns of migration (e.g., seasonal, nomadic, cross-border), and health-related consequences among mobile populations; (iv) Strategies to reach mobile populations including tailored approaches used to overcome mobility-related barriers.

In line with the review objectives, both qualitative and quantitative data were extracted and analyzed. Specific data fields focused on mobile populations (e.g., demographic details, socio-economic status, healthcare access), MDA interventions (e.g., delivery methods, coverage rates, community engagement strategies, period), and effectiveness outcomes (e.g., treatment compliance, reduction in disease prevalence or adverse effects). Discrepancies between reviewers were resolved through discussion or by consulting a third reviewer when necessary. All procedures and rules for data extraction were documented and securely stored with the protocol and extraction datasheet at the research data storage facility of ICER-Mali, based at the University of Sciences, Techniques and Technologies of Bamako (USTTB), Mali.

### Assessment of study quality

In line with the methodological framework for scoping reviews by Arksey & O’Malley (2005), we did not conduct a formal critical appraisal of the included studies, as our objective was to map the extent, range, and nature of the existing evidence rather than attempt to assess certainty in these results or synthesize these in such a way as we would in systematic reviews [28]. Although a formal critical appraisal was not conducted, reviewers noted basic methodological characteristics (e.g., study design, clarity of objectives, and relevance to the review question) during data extraction to support interpretation of the findings.

### Synthesis and presentation of results

Extracted data were summarized using tables and descriptive text. The findings were presented descriptively, highlighting the key characteristics of the included studies, particularly in relation to the populations they examined. Study characteristics were synthesized in a descriptive manner, with a focus on mapping key concepts, populations, and contextual features. Data were organized and displayed following the JBI guidance. We reported data using the PRISMA-ScR guidelines [22], through narrative descriptions and tables.

### Ethics statement

Ethical clearance was obtained from the University of Sciences, Techniques, and Technologies of Bamako’s Ethical Review Committee (Approval No. 2023/11/CE/USTTB), Mali, and from the Health Sciences and Sciences Research Ethics Board of the University of Ottawa (Approval No. H- 02- 23- 8759). This review was conducted as part of a PhD research project. The data included in this review rely on studies already published or in the public domain and therefore do not require ethical approval. The study team respected all procedures of scientific integrity of conducting a scoping review, as mentioned above.

## Results

### Characteristics and summary of included studies

**Fig. 1:**
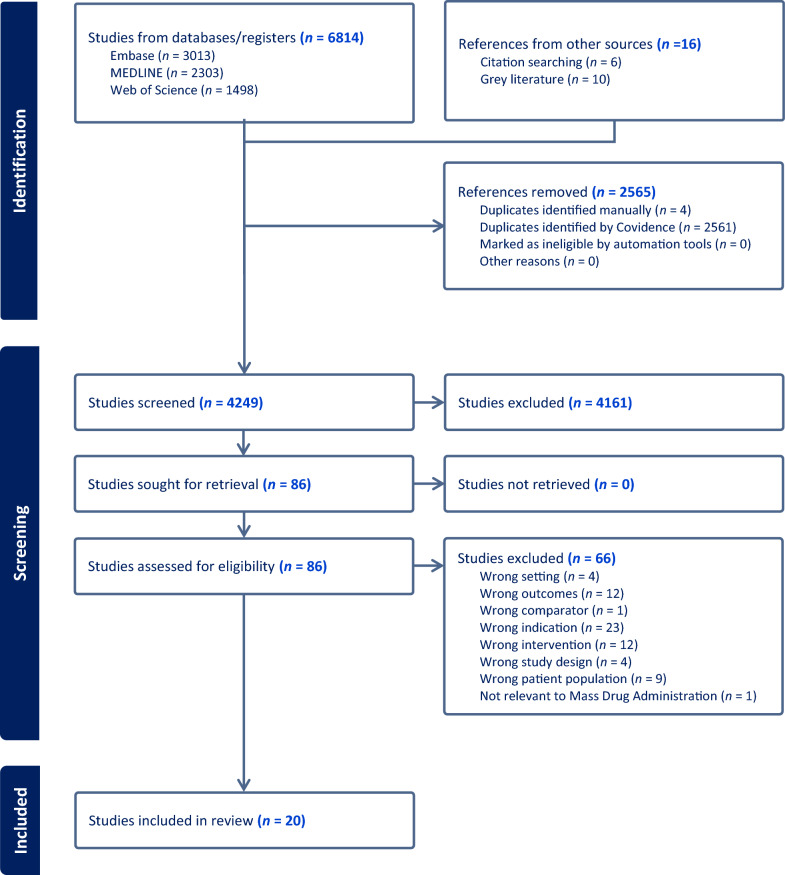
Preferred reporting items for systematic reviews and meta-analysis protocols flow chart

Our search across multiple databases identified a total of 6814 potential studies. Electronic searches were conducted in MEDLINE (Ovid) (*n* = 2303), EMBASE (*n* = 3013), Web of Science (*n* = 1498), Google search (*n* = 16), the WHO search engine (*n* = 5), NGO and technical/financial websites. (see Fig. 1: Preferred reporting items for systematic reviews and meta-analysis protocols flow chart).

After title and abstract screening, only 84 studies met the criteria for full text review. Next, we conducted a comprehensive review of those articles (that met the first step criteria), which resulted in the selection of 20 studies for this scoping review. A detailed overview of the included studies is presented in Table 2.

**Table 2:** Characteristics of included studies. *PC-NTDs* Preventive chemotherapy NTDs

Author (Year)	Title	Country/ Region	Study design	Age of study population	Target neglected tropical diseases	Type of mobility	Citation
Adams et al. (2022)	Leaving no one behind: targeting mobile and migrant populations with health interventions for disease elimination-a descriptive systematic review	Africa (multiple regions)	Descriptive systematic review	Both sexes (Youth and adult)	PC-NTDs malaria, trypanosomiasis, polio, smallpox & rinderpest	Cross-border, seasonal, occupational, displacement	[1]
Ajakaye et al. (2022)	Contrasting epidemiology of urogenital schistosomiasis among pastoral communities surrounding three Ramsar wetland in Nigeria	Nigeria (pastoral communities around Ramsar wetlands)	Cross-sectional study	Both sexes, ages (≤ 10 and ≥ 20)	Schistosomiasis (*S. haematobium*)	Transhumant and seasonal movement with livestock	[2]
Baayenda et al. (2023)	'Follow the cattle': a joint cross-border trachoma MDA perspective	Tanzania, Kenya and Uganda (East Africa)	Descriptive case study	Sex: not reported, Age: Children aged 1–9, Adults ≥ 15 years	Trachoma	seasonal and Transhumant cross-border movement following cattle	[3]
Badia-Rius et al. (2024)	Impact of conflict on the elimination targets of lymphatic filariasis, schistosomiasis and soil-transmitted helminths in Cabo Delgado province, Mozambique	Mozambique (Cabo Delgado province)	Desk-based secondary data analysis	Sex: all genders Age: children and adults	Lymphatic filariasis, schistosomiasis and soil-transmitted helminths	The focus is on forced mobility population and Internal displacement	[4]
Sangare et al. (2024)	Understanding the barriers and facilitators related to never treatment during mass drug administration among mobile and migrant populations in Mali: a qualitative exploratory study	Mali (Tominian, Kalabancoro)	Qualitative exploratory study (in-depth interviews and focus group discussions)	Both sexes (Youth and adult)	Schistosomiasis (context of MDA)	Seasonal migration for agriculture, circular migration for periodic urban work, & displaced migration due to conflict within the same year	[5]
Masong et al. (2021)	Achieving equity in UHC interventions: who is left behind by neglected tropical disease programmes in Cameroon?	Cameroon	Qualitative study applying ethnographic observations	Both sexes (adults eligible for MDA)	Schistosomiasis	Seasonal and cross-border Mobility, Labour/Economic Migration, short-term mobility Rural/urban migration,	[6]
Dorkenoo et al. (2021)	Monitoring migrant groups as a post-validation surveillance approach to contain the potential reemergence of lymphatic filariasis in Togo	Togo (national; border points)	Cross-sectional: programmatic surveillance evaluation (post-validation)	Both sexes (Youth and adult)	Lymphatic filariasis	Cross-border movement of migrants and traders	[7]
Silumbwe et al. (2017)	A systematic review of factors that shape implementation of mass drug administration for lymphatic filariasis in sub-Saharan Africa	Sub‑Saharan Africa (multiple countries)	Systematic review	Both sexes, age not specified	This paper reviews the literature on Lymphatic filariasis elimination	Varied (seasonal, remote, peri-urban)	[8]
Vegvari et al. (2019)	Human population movement can impede the elimination of soil-transmitted helminth transmission in regions with heterogeneity in mass drug administration coverage and transmission potential between villages: a metapopulation analysis	Sub‑Saharan Africa (multiple countries)	Metapopulation transmission modeling analysis	Children (0–15 years-old) Young adults (15–35 years-old) Sex is not explicitly mentioned	Soil-transmitted helminths	Seasonal mobility, Inter-village human movement	[9]
Durrans et al. (2019)	"Moving like birds": a qualitative study of population mobility and health implications in the Bijagos Islands, Guinea Bissau	Guinea‑Bissau (Bijagós Islands)	Qualitative study (interviews and focus group discussions)	Both sexes, age not specified	Lymphatic filariasis, scabies, soil transmitted helminths	Seasonal and circular mobility between islands and mainland	[10]
Gichuki et al. (2024)	Using community-based participatory approaches to improve access to mass drug administration for trachoma elimination in a pastoral conflict area of Kenya	Kenya (Baringo): pastoral conflict area	Mixed methods: community-based participatory research with operational implementation	Both sexes, age not specified	Trachoma	Seasonal mobility, Transhumant and security-driven movement	[11]
Nditanchou et al. (2023)	Ivermectin and doxycycline treatments against Onchocerciasis: adaptations and impact among semi-nomadic population in Massangam Health District, Cameroon	Cameroon (Massangam Health District)	quasi-experimental design: program adaptation evaluation	Both sexes, ages (9–40 et plus)	Onchocerciasis	Seasonal and circular movement	[12]
Mtuy et al. (2021)	Understanding hard-to-reach communities: local perspectives and experiences of trachoma control among the pastoralist Maasai in northern Tanzania	Tanzania (northern Maasai areas)	Qualitative study (Ethnographic research)	Both sexes, age (15–50)	Trachoma (ocular infection caused by *Chlamydia trachomatis*)	Seasonal mobility: transhumant within and across districts	[13]
West et al. (2015)	Risk of Infection with Chlamydia trachomatis from Migrants to Communities Undergoing Mass Drug Administration for Trachoma Control	Tanzania	Longitudinal study (Epidemiological risk analysis)	Children under 10 years old (both sexes)	Trachoma	In-migration from endemic areas	[14]
Harvey et al. (2022)	How can the neglected tropical disease community be inclusive and equitable in program delivery? Reaching refugees and internally displaced people through integrating a 'leave no one behind' approach	Sub‑Saharan Africa (multiple countries: (humanitarian/refugee settings)	Cross-sectional study Programmatic perspective (Leave No One Behind)	Age/sex not reported, interventions targeted eligible children & adults at risk of NTDs	PC-NTDs: trachoma, lymphatic filariasis, schistosomiasis, and soil-transmitted helminths,	Forced displacement and camp-based mobility	[15]
Bush et al. (2018)	Cross-border issues: an important component of onchocerciasis elimination programmes	Cross-border onchocerciasis zones (West and Central Africa)	Programmatic review/commentary	Both sexes, age not specified	Onchocerciasis	Cross-border daily and seasonal movement	[16]
Sanders et al. (2019)	Prevalence of trachoma within refugee camps serving South Sudanese refugees in White Nile State, Sudan: results from population-based surveys	Sudan (White Nile State refugee camps)	Cross-sectional: population-based prevalence surveys	Both sexes, ages (1– 70 et plus)	Trachoma	Forced displacement; camp-to-host movement	[17]
Seck et al. (2023)	Pastoralists residing in northern Senegal	Senegal	Cross-sectional study:	Both sexes, ages (0– 50 et plus)	Schistosomiasis	Transhumant and seasonal movement	[18]
Perez-Saez et al. (2015)	A Theoretical analysis of the geography of schistosomiasis in Burkina Faso highlights the roles of human mobility and water resources development in disease transmission	Burkina Faso	Secondary analysis: geospatial analysis (quantitative techniques)	Not mentioned	Schistosomiasis	Seasonal, circular migration, and local movement between villages and water bodies	[19]
Ervin et al. (2016)	Surveillance and azithromycin treatment for newcomers and travelers’ evaluation (ASANTE) Trial: design and baseline characteristics	Tanzania	Cluster-randomized community trial (baseline survey and intervention design)	Both sexes, ages (1–9) [1–9]	Trachoma	In-migration, seasonal mobility, short-term travel	[20]

Data from the included studies were extracted into an Excel file created for this purpose. The included articles are summarized in Table 3. The articles focused on mobile populations of all ages in Africa, including children and adults eligible for MDA of PC-NTDs. Interventions were delivered in various settings, such as schools, health centers, communities. The selected studies examined four key themes: (1) mobility drivers and challenges of delivering MDA to mobile populations; (2) impact of mobility on MDA coverage; (3) successful strategies for improving MDA; and (4) key recommendations including gaps and proposed solutions for future MDA research.

**Table 3:** Summary of results of all included studies

Author (Year)	Population and setting	Type of intervention	Reasons for mobility	Impact of mobility on MDA coverage and effectiveness	Barriers identified	Facilitators / enabling factors	Key findings relevant to mobility & MDA
Adams et al. (2022)	Mobile and migrant populations (MMP) across elimination programs (Nomadic pastoralists, Migrant laborers, Internally Displaced Persons (IDPs) and refugees)	Mass Drug Administration (MDA) for neglected tropical diseases (NTDs), Vaccination campaigns Vector control interventions, Health education Cross-border coordination for synchronized MDA and surveillance Community health worker mobilizations	Livelihood, conflict, environmental change, labor migration	MMPs are missed by conventional campaigns due to their absence during fixed MDA periods, leading to gaps in coverage and persistent transmission Campaigns timed outside of peak settlement periods may miss entire subgroups; for instance, livestock herders, forest workers, or miners may only be present in certain seasons. Movement between countries can result in “importation” or re-introduction of disease into areas that had achieved control/elimination	Geographic mobility/remoteness, Population enumeration/sampling/timing: seasonal or occupational migration leads to absence during campaigns Language and cultural barriers, mistrust of health systems, misinformation, lower health literacy.Lack of tailored intervention/delivery strategies; most are designed for stable populations	Community engagement; tailored awareness campaigns; side-effect management; flexibility in implementation	The review found that MMP are consistently underserved by elimination programs, with structural, linguistic, and legal barriers driving lower coverage; programs that mapped mobility, synchronized activities across borders, adapted delivery modalities, and engaged trusted intermediaries achieved better reach and equity
Ajakaye et al. (2022)	Pastoralist communities living around three Ramsar wetlands (nomadic livestock herders)	Study focused on epidemiological survey and diagnosis (urine filtration/microscopy for Schistosoma eggs) No recent mass drug administration (MDA) of praziquantel reported in some communities (e.g., no functional health center in Dagona Sanctuary community)	Livelihood (pastoralism), seasonal grazing and water access	Disrupted planning and reduced effectiveness Transhumant mobility around wetlands increases exposure and leads to missed fixed‑site activities; scheduling MDA around pastoral routes and seasons is required to reach these groups	Lack of functional health facilities and recent mass drug administration programs in some communities (Dagona Sanctuary) Insecurity and conflict in some regions affect delivery of interventions Lack of inclusion of pastoral communities in control programs Ecological factors affecting snail vectors (variation between wetlands)	Targeted case finding and MDA scheduling around pastoral calendars; water, sanitation and behavior change interventions	The study reported contrasting, often high, urogenital schistosomiasis prevalence linked to pastoral mobility and water contact patterns, underscoring the need to align MDA and health education with transhumance schedules and to improve access to safe water
Baayenda et al. (2023)	Nomadic pastoralist communities and mobile cattle herders in cross border areas: Ateker ethnic groups along the Uganda-Kenya border Maasai pastoralists along the Kenya-Tanzania border	Synchronized MDA: Cross-border coordination: Simultaneous MDA campaigns in Kenya, Uganda, and Tanzania Community engagement: Involvement of cultural/ kraal leaders for mobilization. Joint planning/ supervision: Shared data, resources, and timelines	Pasture and water availability; seasonal movement	Missed populations: Cross‑border pastoral mobility can undermine isolated national campaigns Recrudescence: Uncoordinated MDA allows reinfection across borders Binational/coordinated MDA scheduling along cattle routes sustains coverage among mobile herders	Asynchronous national schedules; population flux; weak cross-border data sharing. Uncoordinated MDA efforts risk perpetuating trachoma and other NTDs due to gaps in coverage, particularly affecting nomadic populations. Additionally, limited focus on cross-border MDAs and funding restrictions imposed by partners hinders efforts to reach vulnerable groups	Binational coordination; aligning MDA timing/routes to transhumance corridors; joint microplanning	The paper argues that synchronizing trachoma MDA along transhumance routes and sharing data across borders increases access for mobile pastoralists and reduces missed populations
Badia-Rius et al. (2024)	Conflict-affected resident and internally displaced populations	Standard MDA with adapted delivery	Armed conflict and insecurity: Forced mobility from conflict fragments catchment populations and lowers MDA reach unless flexible,	IDPs and nomadic groups were excluded from MDA Displacement may reintroduce parasites to cleared areas Transmission Assessment Surveys (TAS) postponed due to insecurity	Population displacement: Internally displaced persons (IDPs) are often missed during MDA campaigns due to their constant movement Geographic inaccessibility: Approximately 41% of districts are classified as “hard-to-reach,” limiting program coverage Funding gaps: Reduced international support has weakened the sustainability of NTD programs	Adaptive delivery with partners; integrating with humanitarian services; flexible timing and microplanning	Conflict substantially disrupted preventive chemotherapy, displacing communities and lowering coverage, and the authors recommend flexible, risk-informed MDA jointly planned with humanitarian actors to protect elimination timelines
Sangare et al. (2024)	Internally Displaced Persons (IDPs), nomads/transhumants, economic migrants, Seasonal migrants	Mass Drug Administration (MDA) for schistosomiasis, using praziquantel The study explored qualitative insights into barriers and facilitators of MDA participation rather than implementing a new intervention	Livelihood, conflict, migration	MDA campaigns often coincide with periods of high mobility (e.g., rainy season for farming or dry season for mining), leading to missed treatments Mobility contributed to "never-treated" clusters, risking disease resurgence and hindering elimination goals Static MDA strategies failed to adapt to dynamic population movements, resulting in coverage gaps	Lack of communication channels and low education levels in rural and mobile populations, Lack of appropriate training and knowledge of CDD, Discrimination, Economic constraints. Seasonal observations on population shifts	Community engagement; tailored awareness campaigns; side‑effect management; flexibility in implementation	Identified why highly mobile populations remained untreated and proposed tailored, flexible strategies using Levesque’s framework to improve equitable MDA access
Masong et al. (2021)	Populations underserved by NTD programs (including urban poor, migrants, nomads, people with disabilities)	Mass Drug Administration (MDA) for schistosomiasis, using praziquantel The study evaluated both school-based (targeting children) and community-based (door-to-door) distribution strategies	Livelihood change, displacement, service-seeking	MDA campaigns often coincided with peak farming seasons (e.g., July for planting, December for holidays), when migrant populations were absent Mobile groups (e.g., Nigerian laborers) were consistently missed, creating reservoirs for disease transmission Static MDA schedules failed to adapt to dynamic migration patterns, reducing effectiveness	Inadequate sensitization campaigns, limits in CDD training around pregnancy and reproductive health; lack of alignment between distribution and community availability and the exclusion of existing formal and informal governance structures	Inclusive planning; disability-sensitive delivery; outreach in informal settlements; multilingual communication	The study identified who is left behind by NTD programs in Cameroon—particularly migrants, urban poor and people with disabilities—and recommends equity-oriented adaptations, including multilingual communication and service design that addresses mobility and accessibility needs
Dorkenoo et al. (2021)	Nomadic Peuhls', refugees because of conflict Migrant groups entering from neighboring endemic areas	Specific delivery	Work, trade, and travel	Timing Mismatch: Migrants were often absent during MDA campaigns. Cross‑border movement can reintroduce LF into validated areas;	Mobility: Nomadic Peuhls' transient presence complicates surveillance and treatment	Border collaboration and targeted rapid tests; integration with port-of-entry screening	Monitoring migrant groups after LF validation was feasible and helped mitigate reintroduction risk, supporting targeted surveillance of cross-border entrants and migrants from endemic areas helps sustain post-MDA gains
Silumbwe et al. (2017)	Migrants and mobile populations targeted for lymphatic filariasis (LF), MDA across SSA	Mass Drug Administration (MDA) for lymphatic filariasis (LF), primarily using albendazole, ivermectin, or diethylcarbamazine citrate (DEC)	Programmatic and contextual factors	Mobility and unregistered migrations made it difficult to estimate target populations, leading to under- or over-supply of drugs Rapid urbanization and population movements resulted in "hard-to-reach" areas with low MDA coverage	Lack of geographical demarcations and unregistered migrations into rapidly urbanizing areas, delayed drug deliveries at both country and community levels, inappropriate drug delivery strategies, limited number of drug distributors	Strong community engagement and supervision; tailored timing; responsive AE management; incentives for CDDs	Across SSA, MDA performance depended on context-sensitive delivery: programs that engaged community‑directed strategies, aligned timing with local calendars, and ensured strong supervision achieved higher coverage
Vegvari et al. (2019)	Populations across borders, (interconnected villages)	Standard MDA with adapted delivery	Work, trade, social links	Movement from untreated villages reduces the probability of elimination, especially in high-prevalence settings	Human mobility	Coordinated, high-coverage, synchronized rounds; targeting high-mobility links	The model shows that human movement between communities with uneven coverage can sustain transmission and impede elimination, highlighting the value of synchronized, high-coverage MDA and attention to highly connected locations
Durrans et al. (2019)	Displaced populations (due to disaster or conflict); emigrants and immigrants; visitors; education travel; and circulatory movement	Standard MDA with adapted delivery	Livelihoods (fishing, farming), caregiving, social obligations	Migrants absent during MDA rounds reduce treatment coverage Mobile individuals bring infections from untreated areas MDA schedules are not aligned with seasonal return periods	Migrants missing MDA campaigns due to absence during distribution Distrust of health programs or traditional beliefs Hard-to-reach islands with limited health infrastructure	Community-led scheduling; mobile outreach; integrating services with travel calendars	Participants described mobility as a way of life that complicates fixed-schedule services; health interventions should adapt to seasonal calendars and transport realities to avoid missing mobile households
Gichuki et al. (2024)	Pastoralists, internally displaced people (IDPs)	Standard MDA with LNOB approach	Pasture, water access, insecurity	Refugees may reside in isolated areas with limited access to public health facilities, and include various at-risk groups such as children, women, older people, and people with disabilities	Treatment rejection, Refugees may reside in isolated areas with limited access to public health facilities and include various at-risk groups such as children, women, older people and people with disabilities	Co-design with elders; security coordination; gender-sensitive messaging	Using CBPR, the team co-created trachoma MDA strategies with pastoralist communities, strengthening trust and access despite insecurity and distance, and improving local participation
Nditanchou et al. (2023)	Semi‑nomadic populations in onchocerciasis foci	Standard MDA with a systematic approach	Pastoralism and trade	Disrupted planning and reduced effectiveness	Mobility, lack of awareness	Tailored timing, bivouac delivery, and combined treatment strategies	Program adaptations for semi‑nomadic groups—including flexible timing and combined ivermectin–doxycycline strategies—were feasible and improved participation and impact indicators
Mtuy et al. (2021)	Pastoralist Maasai communities	Mass Drug Administration (MDA) of azithromycin (*Zithromax*) for trachoma control, part of the SAFE strategy (Surgery, Antibiotics, Facial cleanliness, Environmental improvement)	Livestock care, seasonal grazing	MDA coincided with seasonal migration (dry season), leading to absences of men and some women	Limited community engagement, lack of trust in government programs, and misaligned priorities such as water access and human-animal conflicts hindered MDA responses. Political-economic factors and top-down resource allocation further reduced effectiveness	Engaging traditional leaders; flexible hours and locations; gender-sensitive approaches	Local perspectives highlighted that trachoma control must align with Maasai mobility patterns and social structures; flexible, leader-endorsed delivery is essential to reach remote manyattas
West et al. (2015)	Communities undergoing trachoma MDA with incoming migrants	Standard MDA with adapted delivery	Work and family movement	Re-emergent infection due to migration may slow progress toward elimination	Some migrant families may have been missing if they moved in and out between censuses	Enhanced surveillance; screening/targeting for new arrivals; synchronized coverage across adjacent areas	The study found that migrants from endemic areas posed a measurable risk of reintroducing C. trachomatis into communities undergoing MDA, suggesting the value of monitoring and targeted strategies for newcomers
Harvey et al. (2022)	Refugees (e.g., Cameroonians fleeing conflict into Nigeria) Internally displaced persons (IDPs) within Nigeria and Niger due to conflict, violence, and natural disasters	Standard MDA with adapted delivery	Conflict, disasters, persecution	Mobile populations are often missed in standard MDA rounds due to transient residence Fluctuating camp populations complicate census-taking and coverage estimates	Lack of data on displaced populations' locations and sizes Insecurity in conflict zones limiting access	Joint planning with humanitarian clusters; inclusion in essential service packages; flexible supply chains	The paper recommends integrating NTD programming into humanitarian response to reach refugees and IDPs, emphasizing joint planning, protection-sensitive delivery, and equitable access
Bush et al. (2018)	Cross-border communities with ongoing transmission	Standard MDA with adapted delivery	Trade, social ties, pastoralism	Disrupted planning, MDA coverage reduced and reduced effectiveness	Language differences, Lack of coordination in MDA schedules, the lack of availability or non-release of funds by governments for CB activities, the lack to date of including CB issues in programmatic planning	Binational agreements; joint microplanning; shared entomological/xenomonitoring data	Cross-border movement is a critical driver of persistent onchocerciasis transmission; coordinated binational MDA and data sharing are necessary to achieve elimination
Sanders et al. (2019)	South Sudanese refugees living in camp in White Nile state	Standard MDA with adapted delivery	Conflict and insecurity	Refugees may reside in isolated areas with limited access to public health facilities and include various at-risk groups such as children, women, older people and people with disabilities	not reported	Camp-specific microplanning; integration with WASH and case management	Refugee camps hosted substantial trachoma prevalence, supporting the need for camp-tailored SAFE implementation, including MDA and improved WASH services
Seck et al. (2023)	Nomadic pastoralist	Standard MDA with adapted delivery	Pastoral livelihoods and water access	Disrupted planning and reduced effectiveness	not reported	Mobile outreach and timing with transhumance; targeted screening	Seroprevalence to Schistosoma soluble egg antigen among nomadic pastoralists indicated ongoing exposure, highlighting the need for targeted surveillance and MDA strategies aligned with transhumance patterns
Perez-Saez et al. (2015)	Human mobility in general	N/A	Agricultural activities, fishing, labor migration, seasonal transhumance	Interestingly, intense human mobility was also shown to exacerbate the impact of the building of the dams in the case of high levels of urban contamination and exposure rates Mobility patterns created spatial heterogeneity in exposure and transmission, which would reduce the effectiveness of MDA if programs do not account for seasonal and occupational migration	High spatial heterogeneity of exposure due to mobility; uneven access to preventive chemotherapy if programs are not adapted to movement patterns	Integration of spatial mobility data into program design and resource allocation could optimize targeting to achieve sustainable interruption of transmission	The study demonstrated that both human mobility and water resource development play critical roles in shaping schistosomiasis transmission in Burkina Faso. Seasonal and occupational mobility patterns significantly affect exposure risk, creating hotspots of persistent transmission
Ervin et al. (2016)	Newcomers and Travelers	Standard MDA with adapted delivery	Resettlement, temporary labor, visiting relatives, trade	Disrupted planning and reduced effectiveness, newcomers and travelers were frequently missed by routine MDA,	High turnover of population; difficulty identifying and treating newcomers in time; risk of untreated individuals sustaining transmission	Incorporating surveillance for new arrivals and systematic outreach helped ensure higher treatment equity	The ASANTE trial highlighted that newcomers and travelers were frequently missed by standard community-wide azithromycin MDA, undermining elimination goals for trachoma

### Drivers of mobility, patterns and barriers to MDA delivery

Dynamic mobility patterns amongst the populations in the papers were found to be driven by a range of factors. Livelihood-related movements, such as seasonal migration for agricultural or construction work, traditional gold and mining, and transhumance among pastoralist communities, cause frequent and often unpredictable population shifts in many settings [29, 30]. Studies included in this review found that mobility patterns often fall outside of the fixed timelines and geographic boundaries of planned MDA campaigns, reducing the likelihood that these populations are reached at the necessary time and place.

Mobile populations including nomadic pastoralists, migrant laborers, IDPs, and refugees experience lower MDA coverage compared to settled populations due to their movement patterns as documented by existing studies [5, 31]. Seasonal migration, driven by work opportunities, climate variability, or conflict, further contributes to missed treatments and inconsistent MDA access [7, 32] For example, pastoralist communities face exclusion due to their reliance on cattle movements, which rarely align with fixed MDA campaign schedules [33, 34].

In Cameroon, static MDA schedules failed to adapt to the cyclical migration of Nigerian laborers, creating reservoirs for schistosomiasis transmission [35]. In Nigeria, a study by Ajakaye et al. (2022) found that pastoralists migrating between Ramsar wetlands exhibited significantly higher schistosomiasis prevalence due to missed MDA opportunities. This study also emphasized the difficulty of reaching these mobile populations with MDA targeting schistosomiasis [36]. Similarly, in Burkina Faso, intense human mobility linked to dam construction exacerbated schistosomiasis transmission [37].

Environmental pressures including recurrent droughts, floods, and resource scarcity, particularly in areas that are ecologically vulnerable, force communities to relocate in search of water, grazing lands, or food security [36, 38]. Such movements can be sudden and unexpected, making MDA and other health services delivery even more difficult.

Finally, studies indicate that conflict, insecurity, and forced displacement driven by armed conflicts and political instability contribute to irregular population movements, particularly in border regions and areas of crisis [31, 32, 39, 40]. Displaced populations may reside in informal settlements, refugee camps, or move across districts or national boundaries, making it difficult for NTD programs to identify, register, and follow up with eligible individuals [39, 41]. In Togo, Dorkenoo et al. (2021) identified that the cross-border movement of nomadic Peuhls posed a risk of reintroducing lymphatic filariasis into areas that had achieved validation of elimination, as these mobile groups were frequently missed by surveillance and treatment campaigns [40]. In Mozambique’s Cabo Delgado province, a desk analysis conducted by Badia-Rius et al. (2024) highlighted that conflicts and forced displacement have systematically excluded IDPs from MDA, thereby disrupting the elimination timelines [39]. Similarly, refugee camps in Sudan reported persistent trachoma transmission due to cross-border mobility [41, 42].

Because of these movement patterns, MDA programs can have significant operational challenges in delivering MDA consistently to mobile and migrant populations. These include logistical barriers such as difficulties in timely drug distribution or tracking eligible individuals across borders or informal settlements [40, 43]. In addition, inadequate awareness about MDA campaigns due to limited community engagement and limited health education among transient groups were reported in a few studies [35, 44–46]. In Mali, low education levels and discrimination reduced participation amongst mobile populations, and instances of never treatment were reported due to exclusion from community-based drug distribution [7]. Additional barriers to MDA delivery also included the presence of language and cultural differences, which hindered communication and trust in health programs [42]. A systematic review identified the lack of tailored intervention strategies for mobile groups [43]

### Impact of population mobility on MDA coverage and effectiveness

Population movement significantly hinders the success of MDA initiatives targeting the elimination of NTDs in Africa, as measured by reported coverage and epidemiological coverage [47]. As highlighted by several studies, mobile populations such as nomadic pastoralists, refugees, IDPs, seasonal laborers/workers, and cross-border populations are frequently missed by national MDA programs, leading to inconsistent coverage, ongoing or re-introduction of transmission, and diminished program effectiveness [5, 7, 29, 30, 36, 40, 48, 49]. In pastoralist and nomadic settings, research from Nigeria, Senegal, and Tanzania have documented direct links between mobility and limited MDA reach [36, 48, 49]. These studies show that pastoralist populations exhibit elevated disease burden and lower MDA participation due to constant movements, remote locations, and cultural barriers. Mtuy et al. (2021) in Tanzania, research emphasized how geographic isolation and mistrust created operational hurdles in mobile population groups [48].

Multiple works highlight how mobile groups are structurally excluded due to inadequate planning, poor community engagement, and health system inflexibility [5, 7, 35]. In Mali, seasonal migration for farming and mining led to clusters of never treated individuals, perpetuating schistosomiasis transmission [7]. Similarly, in Cameroon, Nigerian migrant laborers were consistently missed during MDA rounds, creating reservoirs for schistosomiasis [35]. Reaching mobile populations is not only a matter of equity and social justice (which is essential), but also of absolute necessity to protect the gains that have been made. Failure to treat mobile and migrant groups, programs risk persistent reservoirs of infection that can undermine overall progress.

Beyond routine mobility, conflict-driven displacements exacerbated the strain to MDA efforts [31, 39, 41]. For example, Badia-Rius et al. [26] documented disrupted MDA activities in Mozambique’s conflict zones, while Sanders et al. (2019) reported that South Sudanese refugees in Sudan were not included in national treatment registers [41]. In Niger, refugees in isolated settlements faced limited access to MDA due to security threats and logistical gaps [50].

Cross-border movement is another major factor that further impedes MDA success [29, 30, 42]. These papers emphasized those transhumant pastoralists who cross national borders and create gaps in coordination between countries, risking reintroduction of infections into treated zones and delaying regional elimination efforts’ positive impacts. The transhumance of nomadic Peuhls across districts of Togo, coupled with conflicts with local farmers, frequently led to their absence during MDA campaigns, thereby sustaining lymphatic filariasis transmission [40]. Similarly, in West and Central Africa, uncoordinated cross-border MDA schedules resulted in untreated mobile populations [42]. Baayenda et al. (2023) described how uncoordinated national MDA campaigns in Uganda, Kenya, and Tanzania for trachoma were undermined by cross-border mobility of pastoralists, which allowed for reinfection and sustained transmission [29].

The consequence of these operational challenges is the persistence of NTD transmission in both mobile and host communities. For instance, Adams et al. (2022) found that these populations are often missed during fixed campaign periods, resulting in pockets of untreated individuals who sustain transmission cycles [5]. Other studies from Tanzania and Kenya found that migrants were 2.5 times more likely to be infected with trachoma than resident populations, directly contributing to re-emergent infections in communities undergoing MDA [33, 51]. Furthermore, uncoordinated MDA efforts led to re-emergent trachoma infections via migrant reintroduction [51, 52]. Finally, in sub-Saharan Africa, movement from untreated villages appeared to reduce the probability of elimination by 30% in some high-prevalence settings [38].

Studies recommended adapting interventions to the respective cultural and geographical contexts. Gichuki et al. (2024) found that participatory approaches of campaigns improved MDA uptake in Kenya’s conflict areas [33]. Sangare et al. (2024) emphasized aligning MDA campaigns with local movement patterns [7]. Synchronized cross-border MDA in Tanzania, Kenya, and Uganda has been reported to increase access and decrease the number of populations missed [29]. Modeling studies also support the disruptive effect of mobility [32, 36, 38] demonstrating how untreated mobile individuals sustain transmission of soil-transmitted helminths, urging dynamic MDA strategies. These findings emphasize the need for flexible MDA delivery strategies that align with mobility patterns, rather than relying solely on fixed geographical targets. In line with this, the flexible delivery in our discussion explicitly includes integrated drug delivery strategies to optimize coverage and minimize parallel, uncoordinated efforts.

### Successful strategies documented and proposed solutions for future effective MDA

Key strategies have emerged from the evidence to improve MDA outcomes among mobile populations:

- (1) Integrate mobility mapping into MDA microplanning

The paths taken by migrating populations and their settlement patterns should be accurately identified. Multiple studies recommend incorporating real-time mobility data into national MDA policies to better capture missed populations and guide adaptive delivery strategies [5, 38, 40]. For instance, a study documented how cross-border trachoma programs in Uganda, Kenya, and Tanzania mapped pastoralist cattle-migration routes to align MDA campaigns with seasonal population movements [29]. Health teams coordinated between districts and across borders, using local knowledge of cattle camps and migration calendars to ensure that mobile pastoralists received treatment. This approach directly improved MDA reach among highly mobile pastoralist groups, reducing systematic exclusion due to seasonal mobility. This demonstrated the use of field-validated mobility mapping as a microplanning tool [29].

Similarly, Nditanchou et al. (2024) implemented mobility mapping in MDA microplanning, to better improve coverage in hard-to-reach mobile groups. The program adapted treatment delivery to semi-nomadic Fulani herders by mapping seasonal transhumance patterns (where and when herders move with cattle). Community distributors and health workers planned flexible treatment points along migration routes, and timed ivermectin + doxycycline delivery with seasonal presence of nomads. Reported uptake improved, showing that mobility-informed planning can reduce systematic non-treatment [53].

- (2) Enhance cross-border and regional collaboration

Given the transboundary nature of many mobile populations, studies emphasized the importance of harmonized MDA campaigns between neighboring countries, shared surveillance data, and coordinated health policies to prevent reinfection across borders [29, 30, 42]; For example, to work together to eliminate trachoma among cross-border nomadic populations, Kenya, Tanzania, and Uganda have adopted a strategy known as "synchronized MDA." The initiative intends to control *Chlamydia trachomatis* infections by administering antibiotics to the entire population in endemic areas across borders during the same treatment period. The implementation of MDA and TT surgery in April 2019 reached an additional 40,000 people in eight nomadic border regions with MDA thanks to collaborative planning meetings that resulted in successful social mobilization, team selection, and unanimous support from local leaders [29].

A similar collaboration in Tanzania and Kenya MDA in July and December 2022 targeted nomadic Maasai communities along their shared border. Through joint planning, community mobilization, and coordinated supervision, the campaign reached about one million people in five days. This approach overcame the limitations of isolated interventions and marked a shift toward a more coordinated regional strategy for trachoma elimination [29].

- (3) Adopt flexible and decentralized delivery models

Adapting MDA timing and methods to seasonal and cultural calendars is critical. Evidence from Mtuy et al. (2019, 2021), Ajakaye et al. (2022), and Seck et al. (2023) supported using mobile outreach teams, integration with routine services, and alignment with community events to increase participation [34, 36, 48, 49]. In Baringo County, Kenya, a conflict-affected pastoral area, MDA coverage for trachoma remained low (around 68%), below the WHO recommended threshold (80%). The authors implemented a participatory community approach in several phases (pre-intervention, intervention, post-intervention), involving the community in identifying barriers to participation and access to MDA. This approach aimed at adapting mobilization and treatment delivery methods to reflect the realities of the local population, including mobility patterns and specific constraints. As a result, the overall MDA coverage in the area increased from 67.6% in 2021 to 87% in 2023 thus meeting the WHO threshold of 80%. MDA coverage of trachoma and other neglected tropical diseases has improved as a result of the application of community-based, participatory methodologies in the development and implementation of data-driven strategies [33]. Masong et al. (2021) further emphasize that migrant farmers, seasonal workers, out-of-school children, and women of childbearing age were regularly overlooked. They recommend stronger collaboration with local governance and more flexible planning to ensure no population groups are left behind [35].

- (4) Prioritize inclusion and community engagement

Meaningful participation of mobile groups in program design fosters trust and ownership. Kenya's synchronized MDA approach exemplifies the strong involvement of community leaders [29]. Similarly, studies recommend engaging traditional leaders, local health actors, and community representatives to design culturally appropriate and context specific interventions [5, 7, 33].

- (5) Monitor migrant populations in elimination and post-elimination settings

Continuous monitoring is essential to prevent re-emergence. Research in Togo after LF elimination shows the value of migrant-focused surveillance, where seasonal workers, nomadic Peulhs, traders, and cross-border migrants were actively screened to prevent resurgence. This approach complemented Transmission Assessment Surveys (TAS), which primarily focus on resident populations [40]**.**

- (6) Address structural inequities in program design

To ensure equity, NTD programs must explicitly target those at risk of being left behind. Integrating equity frameworks into national NTD policies and strategies can close access gaps for some mobile populations, as demonstrated by some studies [5, 39]. The review by Adams et al. (2022), highlights that although most programs still rely on census-based planning which excludes mobile populations, some projects have adapted through the use of participatory mapping of migration routes, use of GPS/GIS tools, and time-location sampling to reach mobile populations. These remain exceptions rather than standard practice, showing progress but not yet systemic change [5].

## Discussion

We conducted a scoping review to explore current evidence of the impact of mobility on MDA delivery, coverage and effectiveness for NTDs in Africa. The review found that mobility particularly among nomadic pastoralists, seasonal workers, IDPs, and cross-border populations leads to systematic exclusion from MDA campaigns. While some barriers appear universal, effective and documented solutions vary considerably across geographic contexts and types of mobility. The following discussion summarizes these findings, examines their implications for policy and practice, identifies limitations in the existing literature, and proposes directions for future research.

Our analysis identified four main themes: challenges related to mobility, the impact on MDA coverage, effective strategies, and policy recommendations. The reviewed studies consistently indicate that mobile populations have lower MDA coverage than sedentary populations. This exclusion is explained by complex mobility patterns, often driven by economic, environmental, and political factors [5, 7, 32, 50]. For example, transhumant herders are frequently absent during fixed campaigns [33], while those displaced by conflict escape health registries in their districts of origin and are not taken into account in the registrations in their host districts [39]. Similar patterns occur in immunization studies, where mobile populations experience significantly lower rates compared to settled communities [30, 35, 41, 54]. Seasonal migrants frequently miss scheduled immunizations [55], while conflict-displaced populations fall through the gaps between home and host regions [56]. In Kenya, Gamino et al. (2016) reported significantly lower vaccination rates among nomadic children (28%) compared to settled populations (85%) [57]. These disparities in coverage stem from complex mobility dynamics, influenced by factors such as economic hardship, environmental stressors, and political instability [55, 56, 58]. While these examples are drawn from the vaccination literature rather than NTD-specific MDA, they provide relevant parallels. Overall, these findings highlight health campaigns' weakness (including vaccinations and NTD programs) in reaching highly mobile population groups.

The impact of mobility patterns on MDA campaigns varies by the nature of mobility. Pastoral populations, such as transhumant herders in the Sahel, suffer from a temporal misalignment with fixed campaigns [48]. Mobile populations often remain invisible to health systems due to poor enumeration. Many MDA failures are rooted in the systemic lack of data on these groups. These challenges are not unique to MDA campaigns; similar issues appear in measles immunization efforts in Chad, Ghana and northern Kenya, where nomadic and pastoralist groups were repeatedly missed during campaigns because of inadequate tracking [59–61]. Moreover, similar operational barriers have been observed in seasonal malaria chemoprevention campaigns, where mobile nomadic populations were missed because they were often excluded in the enumeration data, thereby reducing the overall effectiveness of interventions [62, 63].

### Implications for policy, practice, and strengthening health systems

The findings of this review have clear implications for operational research, policy, and public health practice. Patterns of barriers and enablers indicate that the main challenges in reaching mobile populations are largely systemic rather than technical, reflecting gaps in implementation rather than gaps in technical knowledge.

Strengthening health system planning, first by addressing the loss of critical health information, is essential. National programs should adopt innovative enumeration strategies, such as mobile phone data analysis, satellite imagery, time-location sampling, and integration with refugee/IDP registries, to make mobile populations visible to health systems. Second, national programs should incorporate mobility data into their microplanning to strengthen their planning systems. Third, real-time mapping tools can help track mobile groups and reduce omissions in reaching these mobile groups. Fourth, cross-border partnerships should be institutionalized, with harmonized protocols, dedicated funding, and permanent health committees to reduce dependence on individual leaders. Additionally, pre- and post-elimination surveillance systems should be adapted to include mobile and migrant groups.

Collectively, these strategies support targeted or integrated approaches to ensure mobile populations are not overlooked in MDA and other public health services. These results indicate that such approaches could substantially improve MDA services, highlighting the need for flexible, integrated drug delivery and community-tailored strategies that can be adapted across different disease control programs.

### Intersectionality of vulnerability

Mobility interacts with other forms of marginalization, including language barriers, gender, socioeconomic status, and cultural norms, increasing health vulnerabilities of these communities. Framing populations as simply "mobile vs. sedentary" risks oversimplifying these complex challenges, face similar exclusions due to intersecting vulnerabilities. Effective strategies, such as the use of trusted intermediaries, multilingual communications, and culturally appropriate services, benefit not only mobile populations but also other hard-to-reach groups. Integrating an intersectional lens into MDA planning and implementation can increase both the relevance and equity impact of campaigns.

### Institutionalizing innovation

The study highlights successful cases of MDA implementation in which exceptional leadership and effective problem solving overcome bureaucratic and/or financial obstacles [29, 43]. However, these successes are often fragile and may not be sustainable, particularly given high turnover among health personnel. Future policy recommendations should focus on institutionalizing these innovations by establishing permanent cross-border committees, providing professional incentives for healthcare workers during campaigns, and implementing integrated, flexible funding mechanisms to reduce reliance on temporary funding support. Such measures can help ensure that effective strategies are sustainable, scalable, and resilient over time.

## Limitations and future research directions

This review has some limitations related both to the evidence extracted and the analysis. We deliberately limited our search to mobile populations and NTDs for which WHO recommends MDA for control and elimination, and to English and French literature published between 2000 and February 2025. Excluding related programs, such as vaccination and seasonal malaria chemoprevention, may have narrowed the scope of relevant insights and reduced opportunities for cross-disease program learning. Most included studies were qualitative or descriptive, with limited use of rigorous quantitative or comparative methods. A limitation of this review is the lack of a structured search of gray literature, which may have resulted in the omission of relevant reports or evaluations that were not captured through our informal approach. As a scoping review, no formal quality assessment was conducted, limiting our ability to comment on the robustness of the included evidence. Few studies provided clear comparisons of MDA coverage between mobile and sedentary populations, restricting generalizability and suggesting potential publication bias. More importantly, no study has yet evaluated the cost-effectiveness of adaptive MDA strategies or quantified the relative contribution of mobility versus other intersecting vulnerabilities. Future research should prioritize rigorous comparative studies to evaluate adaptive MDA strategies, generate robust evidence for policy, and inform program design.

## Conclusions

This review highlights the critical importance of including mobile populations in NTD elimination efforts. While challenges remain significant, the evidence supports a shift from rigid, fixed approaches to more flexible, dynamic, and context-specific strategies tailored to mobile communities. Future steps should include developing mobility and enumeration indicators, testing integrated MDA delivery adapted to movement patterns, and establishing partnerships with other health campaigns, such as immunization. Achieving equitable MDA coverage will require multisectoral coordination, dedicated funding, and political will to balance standardized protocols with locally adapted delivery models. Filling data gaps and addressing the intersectional vulnerabilities of mobile and marginalized populations is vital to achieving NTD elimination. Similarly, institutionalizing innovative approaches to service delivery ensures that successes are sustainable and scalable. Failure to reach these groups could reverse decades of progress. Ensuring that elimination strategies explicitly integrate mobility considerations, intersectional vulnerability, and sustainable innovations will help secure irreversible interruption of transmission.

## Supplementary Information

Supplementary Material 1.

Supplementary Material 2.

Supplementary Material 3.

## Data Availability

The datasets generated and/or analyzed during the current study are available from the corresponding author on reasonable request.

## Abbreviations

ABS: Australian bureau of statistics
CDC: Centers for disease control and prevention
GIS: Geographic information systems
GPELF: Global program for the elimination of lymphatic filariasis
IDPs: Internally displaced persons
JBI: Joanna briggs institute methodology
LF: Lymphatic filariasis
MDA: Mass drug administration
MMPs: Mobile and migrant populations
NTDs: Neglected tropical diseases
PRESS: Peer review of electronic search strategies
PRISMA-P: Preferred reporting items for systematic reviews and meta-analysis protocols
PC-NTDs: Preventive chemotherapy NTDs
PRISMA-ScR: PRISMA extension for scoping reviews
STPH: Swiss tropical and public health institute
ILO: The international labour organization
IOM: The international organization for migration
PROSPERO: The international prospective register of systematic reviews
USAID: The United States agency for international development
UNHCR: United Nations High Commissioner for Refugees
USTTB: Université des Sciences, des Techniques et des Technologies de bamako
WHO: World health organization
