# Supplementary material for: The influence of human population movement on mass drug administration for neglected tropical diseases: a scoping review
Source: Infect Dis Poverty. 2026 Apr 7;15:40. doi: 10.1186/s40249-026-01433-w (PMC13054986; doi:10.1186/s40249-026-01433-w)
Supplement: Supplementary file 1 — Supplementary Material 1. [file 40249_2026_1433_MOESM1_ESM.pdf]

Medline (Ovid)
Ovid MEDLINE(R) ALL <1946 to February 19, 2025>

#	Query	Results from 20 Feb 2025
1	exp Population Dynamics/	86,319
2	population dynamic*.ti,ab,kf.	42,190
3	exp "Emigration and Immigration"/ or exp "Emigrants and Immigrants"/ or exp "Transients and Migrants"/	53,462
4	(Migrant? or migration? or Transient? or Emigrant? or Immigrant? or refugee?).ti,ab,kf.	769,316
5	exp Human Migration/	28,352
6	Refugees/ or Refugee Camps/	14,614
7	Cross-border population*.ti,ab,kf.	29
8	(Internally displaced adj2 (person* or people or population)).ti,ab,kf.	863
9	(pastoral* or nomad* or transhum*).ti,ab,kf.	7,918
10	Travel pattern*.ti,ab,kf.	469
11	("no one behind" or ("no one" adj2 "left behind")).ti,ab,kf.	403
12	or/1-11	868,090
13	exp Filariasis/ or exp Elephantiasis, Filarial/ or exp Mass Drug Administration/ or exp Albendazole/	21,599
14	(Drug distribution campaign or community-based treatment or (mass adj2 (administration* or treatment)) or NTD program* or MDA adherence or MDA uptake or MDA coverage).ti,ab,kf.	6,447
15	exp Trachoma/ or exp Ivermectin/ or exp Onchocerciasis/ or exp Schistosomiasis/ or exp Anthelmintics/	99,921
16	(Mass Chemotherap* or Preventive chemotherap*).ti,ab,kf.	1,051
17	(Health adj2 (intervention* or system?)).ti,ab,kf.	201,341
18	(Health equity or (access adj1 healthcare) or healthcare barrier* or (health adj2 system strengthening) or implementation).ti,ab,kf.	430,360
19	exp Neglected Diseases/ or exp Helminthiasis/ or exp Schistosomiasis/ or exp Tropical Medicine/	145,504
20	(Disease? adj2 (elimination or control or eradication)).ti,ab,kf.	87,970
21	Neglected Tropical disease?.ti,ab,kf.	6,485
22	(Lymphatic filariasis* or Bancroftian filariasis or Brugia malayi or Brugia timori or Filarial lymphedema or Wuchereria bancrofti).ti,ab,kf.	6,506
23	(Onchocerciasis* or river blindness or onchocerca volvulus).ti,ab,kf.	5,290

24	(Schistosomiasis* or Bilharziasis or Snail fever or Schistosome or Schistosoma haematobium or (Schistosoma adj2 (mansoni or japonicum or mekongi or intercalatum))).ti,ab,kf.	33,328
25	(Soil-transmitted helminth* or Geohelminths or Intestinal helminths or Hookworm infection or Ankylostomose or Ancylostoma duodenale or Necator americanus or Ascariasis or Ascaris lumbricoides or Trichuriasis or Trichuris trichiura or Roundworm infection).ti,ab,kf.	11,871
26	(Trachoma* or ("Chronic conjunctivitis" and "Chlamydia trachomatis")).ti,ab,kf.	20,114
27	Parasitic infection*.ti,ab,kf.	11,226
28	Vector-borne disease*.ti,ab,kf.	6,100
29	or/13-28	925,872
30	Africa/ or exp Africa South of the Sahara/	308,626
31	(africa or Angola or Congo or Ghana or Mauritius or Sierra Leone or Benin or Cote d'Ivoire or Guinea or Somalia or Botswana or Guinea-Bissau or Mozambique or South Africa or Burkina Faso or Djibouti or Kenya or Namibia or Sudan or Burundi or Equatorial Guinea or Lesotho or Niger or Cabo Verde or Eritrea or Liberia or Nigeria or Togo or Cameroon or Eswatini or Madagascar or Rwanda or Tanzania or Central African or Ethiopia or Malawi or Sao Tome or Uganda or Chad or Gabon or Mali or Senegal or Zambia or Comoros or Gambia or Mauritania or Seychelles or Zimbabwe).ti,ab,kf.	500,552
32	30 or 31	574,619
33	12 and 29 and 32	3,108
34	limit 33 to (english or french)	2,973
35	limit 34 to yr="2000 -Current"	2,293

exp Population Dynamics/
population dynamic*.ti,ab,kf.
exp "Emigration and Immigration"/ or exp "Emigrants and Immigrants"/ or exp "Transients and Migrants"/
(Migrant? or migration? or Transient? or Emigrant? or Immigrant? or refugee?).ti,ab,kf.
exp Human Migration/
Refugees/ or Refugee Camps/
Cross-border population*.ti,ab,kf.
(Internally displaced adj2 (person* or people or population)).ti,ab,kf.
(pastoral* or nomad* or transhum*).ti,ab,kf.
Travel pattern*.ti,ab,kf.
("no one behind" or ("no one" adj2 "left behind")).ti,ab,kf.
or/1-11
exp Filariasis/ or exp Elephantiasis, Filarial/ or exp Mass Drug Administration/ or exp

Albendazole/
(Drug distribution campaign or community-based treatment or (mass adj2 (administration* or treatment)) or NTD program* or MDA adherence or MDA uptake or MDA coverage).ti,ab,kf.
exp Trachoma/ or exp Ivermectin/ or exp Onchocerciasis/ or exp Schistosomiasis/ or exp Anthelmintics/
(Mass Chemotherap* or Preventive chemotherap*).ti,ab,kf.
(Health adj2 (intervention* or system?)).ti,ab,kf.
(Health equity or (access adj1 healthcare) or healthcare barrier* or (health adj2 system strengthening) or implementation).ti,ab,kf.
exp Neglected Diseases/ or exp Helminthiasis/ or exp Schistosomiasis/ or exp Tropical Medicine/
(Disease? adj2 (elimination or control or eradication)).ti,ab,kf.
Neglected Tropical disease?.ti,ab,kf.
(Lymphatic filariasis* or Bancroftian filariasis or Brugia malayi or Brugia timori or Filarial lymphedema or Wuchereria bancrofti).ti,ab,kf.
(Onchocerciasis* or river blindness or onchocerca volvulus).ti,ab,kf.
(Schistosomiasis* or Bilharziasis or Snail fever or Schistosome or Schistosoma haematobium or (Schistosoma adj2 (mansoni or japonicum or mekongi or intercalatum))).ti,ab,kf.
(Soil-transmitted helminth* or Geohelminths or Intestinal helminths or Hookworm infection or Ankylostomose or Ancylostoma duodenale or Necator americanus or Ascariasis or Ascaris lumbricoides or Trichuriasis or Trichuris trichiura or Roundworm infection).ti,ab,kf.
(Trachoma* or ("Chronic conjunctivitis" and "Chlamydia trachomatis")).ti,ab,kf.
Parasitic infection*.ti,ab,kf.
Vector-borne disease*.ti,ab,kf.
or/13-28
Africa/ or exp Africa South of the Sahara/
(africa or Angola or Congo or Ghana or Mauritius or Sierra Leone or Benin or Cote d'Ivoire or Guinea or Somalia or Botswana or Guinea-Bissau or Mozambique or South Africa or Burkina Faso or Djibouti or Kenya or Namibia or Sudan or Burundi or Equatorial Guinea or Lesotho or Niger or Cabo Verde or Eritrea or Liberia or Nigeria or Togo or Cameroon or Eswatini or Madagascar or Rwanda or Tanzania or Central African or Ethiopia or Malawi or Sao Tome or Uganda or Chad or Gabon or Mali or Senegal or Zambia or Comoros or Gambia or Mauritania or Seychelles or Zimbabwe).ti,ab,kf.
30 or 31
12 and 29 and 32
limit 33 to (english or french)
limit 34 to yr="2000 -Current"

https://login.proxy.bib.uottawa.ca/login?url=http://ovidsp.ovid.com/ovidweb.cgi?T=JS&NEWS=N&PAGE=main&SHAREDSEARCHID=1CqcoPrieOK5CrW85VooEwRjvdfs9zSJqQVhEEhOgpenAiaV5qPijFk7088xHgKuk

EMBASE

Embase Classic+Embase <1947 to 2025 February 19>

#	Query	Results from 20 Feb 2025
1	population dynamics/	60,175
2	population dynamic*.ti,ab,kf.	18,680
3	migration/ or forced migration/ or immigration/ or exp migrant/	104,166
4	(Migrant? or migration? or Transient? or Emigrant? or Immigrant? or refugee?).ti,ab,kf.	975,600
5	refugee/ or asylum seeker/ or refugee camp/	20,748
6	Cross-border population*.ti,ab,kf.	36
7	(Internally displaced adj2 (person* or people or population)).ti,ab,kf.	929
8	(pastoral* or nomad* or transhum*).ti,ab,kf.	8,681
9	Travel pattern*.ti,ab,kf.	498
10	("no one behind" or ("no one" adj2 "left behind")).ti,ab,kf.	437
11	or/1-10	1,074,514
12	exp filariasis/ or exp lymphatic filariasis/ or exp onchocerciasis/ or elephantiasis/ or albendazole/ or mass drug administration/	43,706
13	(Drug distribution campaign or community-based treatment or (mass adj2 (administration* or treatment)) or NTD program* or MDA adherence or MDA uptake or MDA coverage).ti,ab,kf.	9,045
14	trachoma/ or ivermectin/ or exp schistosomiasis/ or exp onchocerciasis/	62,096
15	(Mass Chemotherap* or Preventive chemotherap*).ti,ab,kf.	1,368
16	(Health adj2 (intervention* or system?)).ti,ab,kf.	258,940
17	(Health equity or (access adj1 healthcare) or healthcare barrier* or (health adj2 system strengthening) or implementation).ti,ab,kf.	555,839
18	neglected disease/ or tropical medicine/ or helminthiasis/	37,548
19	(Disease? adj2 (elimination or control or eradication)).ti,ab,kf.	125,979
20	Neglected Tropical disease?.ti,ab,kf.	7,913
21	(Lymphatic filariasis* or Bancroftian filariasis or Brugia malayi or Brugia timori or Filarial lymphedema or Wuchereria bancrofti).ti,ab,kf.	8,130
22	(Onchocerciasis* or river blindness or onchocerca volvulus).ti,ab,kf.	6,217
23	(Schistosomiasis* or Bilharziasis or Snail fever or Schistosome or Schistosoma haematobium or (Schistosoma adj2 (mansoni or japonicum or mekongi or intercalatum))).ti,ab,kf.	39,667
24	(Soil-transmitted helminth* or Geohelminths or Intestinal helminths or Hookworm infection or Ankylostomose or Ancylostoma duodenale or	14,192

	Necator americanus or Ascariasis or Ascaris lumbricoides or Trichuriasis or Trichuris trichiura or Roundworm infection).ti,ab,kf.	
25	(Trachoma* or ("Chronic conjunctivitis" and "Chlamydia trachomatis")).ti,ab,kf.	26,074
26	Parasitic infection*.ti,ab,kf.	14,495
27	Vector-borne disease*.ti,ab,kf.	6,905
28	or/12-27	1,084,982
29	africa/ or exp "africa south of the sahara"/	416,819
30	(africa or Angola or Congo or Ghana or Mauritius or Sierra Leone or Benin or Cote d'Ivoire or Guinea or Somalia or Botswana or Guinea-Bissau or Mozambique or South Africa or Burkina Faso or Djibouti or Kenya or Namibia or Sudan or Burundi or Equatorial Guinea or Lesotho or Niger or Cabo Verde or Eritrea or Liberia or Nigeria or Togo or Cameroon or Eswatini or Madagascar or Rwanda or Tanzania or Central African or Ethiopia or Malawi or Sao Tome or Uganda or Chad or Gabon or Mali or Senegal or Zambia or Comoros or Gambia or Mauritania or Seychelles or Zimbabwe).ti,ab,kf.	609,944
31	29 or 30	692,212
32	11 and 28 and 31	3,495
33	limit 32 to (english or french)	3,383
34	limit 33 to yr="2000 -Current"	2,986

population dynamics/
population dynamic*.ti,ab,kf.
migration/ or forced migration/ or immigration/ or exp migrant/
(Migrant? or migration? or Transient? or Emigrant? or Immigrant? or refugee?).ti,ab,kf.
refugee/ or asylum seeker/ or refugee camp/
Cross-border population*.ti,ab,kf.
(Internally displaced adj2 (person* or people or population)).ti,ab,kf.
(pastoral* or nomad* or transhum*).ti,ab,kf.
Travel pattern*.ti,ab,kf.
("no one behind" or ("no one" adj2 "left behind")).ti,ab,kf.
or/1-10
exp filariasis/ or exp lymphatic filariasis/ or exp onchocerciasis/ or elephantiasis/ or albendazole/ or mass drug administration/
(Drug distribution campaign or community-based treatment or (mass adj2 (administration* or treatment)) or NTD program* or MDA adherence or MDA uptake or MDA coverage).ti,ab,kf.
trachoma/ or ivermectin/ or exp schistosomiasis/ or exp onchocerciasis/
(Mass Chemotherap* or Preventive chemotherap*).ti,ab,kf.
(Health adj2 (intervention* or system?)).ti,ab,kf.

(Health equity or (access adj1 healthcare) or healthcare barrier* or (health adj2 system strengthening) or implementation).ti,ab,kf.
neglected disease/ or tropical medicine/ or helminthiasis/
(Disease? adj2 (elimination or control or eradication)).ti,ab,kf.
Neglected Tropical disease?.ti,ab,kf.
(Lymphatic filariasis* or Bancroftian filariasis or Brugia malayi or Brugia timori or Filarial lymphedema or Wuchereria bancrofti).ti,ab,kf.
(Onchocerciasis* or river blindness or onchocerca volvulus).ti,ab,kf.
(Schistosomiasis* or Bilharziasis or Snail fever or Schistosome or Schistosoma haematobium or (Schistosoma adj2 (mansoni or japonicum or mekongi or intercalatum))).ti,ab,kf.
(Soil-transmitted helminth* or Geohelminths or Intestinal helminths or Hookworm infection or Ankylostomose or Ancylostoma duodenale or Necator americanus or Ascariasis or Ascaris lumbricoides or Trichuriasis or Trichuris trichiura or Roundworm infection).ti,ab,kf.
(Trachoma* or ("Chronic conjunctivitis" and "Chlamydia trachomatis")).ti,ab,kf.
Parasitic infection*.ti,ab,kf.
Vector-borne disease*.ti,ab,kf.
or/12-27
africa/ or exp "africa south of the sahara"/
(africa or Angola or Congo or Ghana or Mauritius or Sierra Leone or Benin or Cote d'Ivoire or Guinea or Somalia or Botswana or Guinea-Bissau or Mozambique or South Africa or Burkina Faso or Djibouti or Kenya or Namibia or Sudan or Burundi or Equatorial Guinea or Lesotho or Niger or Cabo Verde or Eritrea or Liberia or Nigeria or Togo or Cameroon or Eswatini or Madagascar or Rwanda or Tanzania or Central African or Ethiopia or Malawi or Sao Tome or Uganda or Chad or Gabon or Mali or Senegal or Zambia or Comoros or Gambia or Mauritania or Seychelles or Zimbabwe).ti,ab,kf.
29 or 30
11 and 28 and 31
limit 32 to (english or french)
limit 33 to yr="2000 -Current"

https://login.proxy.bib.uottawa.ca/login?url=http://ovidsp.ovid.com/ovidweb.cgi?T=JS&NEWS=N&PAGE=main&SHAREDSEARCHID=4kev5FfddXrQB4UnxSVlz0WLHneBX7LjbIH8PSvou1CJgdk4gj6ZE7Gc5MWpxCtqy

WEB OF SCIENCE

Lien pour lancer la recherche dans Web-of-Science : obtenir 1487 articles à exporter
https://www.webofscience.com/wos/woscc/summary/a06a0336-ffbb-4946-a4ee-85c2e7c90bce-014c4d95f9/relevance/1

Détail de la recherche :

TS=((((population$ NEAR/1 dynamic*) or migrant* or migration? or transient? or emigrant* or immigrant* or refugee$ or (Cross-border NEAR/1 population$) or (“Internally displaced” NEAR/2 (person* or people or population$)) or pastoral* or nomad* or transhum* or travel NEAR/1 pattern* or "no one behind" or ("no one" NEAR/2 "left behind")))) AND (((“Drug distribution campaign” or “community-based treatment” or (mass NEAR/2 (administration* or treatment)) or NTD program* or MDA adherence or MDA uptake or MDA coverage or ((mass or preventive) NEAR/1 chemotherap*) or (Health NEAR/2 (intervention* or system?)) or “Health equity” or (access NEAR/1 healthcare) or (healthcare NEAR/1 barrier*) or (health NEAR/2 (“system strengthening” or implementation)))) OR ((((Disease$ NEAR/2 (elimination or control or eradication)) or ((Neglected or Tropical) NEAR/1 disease$) or “Lymphatic filariasis” or “Bancroftian filariasis” or “Brugia malayi” or “Brugia timori” or “Filarial lymphedema” or “wuchereria bancrofti” or Onchocerciasis* or “river blindness” or “onchocerca volvulus” or trachoma or Ivermectin or onchocerciasis or anthelmintics or Filariasis or Filarial or Elephantiasis or Albendazole or Helminthiasis or Schistosom* or Bilharziasis or “Snail fever” or (Schistosoma NEAR/2 (mansoni or japonicum or mekongi or intercalatum or haematobium))))) OR (((Soil-transmitted NEAR/1 helminth*) or Geohelminths or “Intestinal helminths” or “Hookworm infection” or Ankylostomose or “Ancylostoma duodenale” or “Necator americanus” or Ascariasis or “Ascaris lumbricoides” or Trichuriasis or “Trichuris trichiura” or “Roundworm infection” or Trachoma* or "Chronic conjunctivitis" and "Chlamydia trachomatis" or (Parasitic* NEAR/1 infection*) or (Vector-borne NEAR/1 disease*)))) AND ((africa or Angola or Congo or Ghana or Mauritius or Sierra Leone or Benin or Cote d'Ivoire or Guinea or Somalia or Botswana or Guinea-Bissau or Mozambique or South Africa or Burkina Faso or Djibouti or Kenya or Namibia or Sudan or Burundi or Equatorial Guinea or Lesotho or Niger or Cabo Verde or Eritrea or Liberia or Nigeria or Togo or Cameroon or Eswatini or Madagascar or Rwanda or Tanzania or Central African or Ethiopia or Malawi or Sao Tome or Uganda or Chad or Gabon or Mali or Senegal or Zambia or Comoros or Gambia or Mauritania or Seychelles or Zimbabwe)) AND (LA==("ENGLISH" OR "FRENCH"))) AND (PY==("2025" OR "2024" OR "2023" OR "2022" OR "2021" OR "2020" OR "2019" OR "2018" OR "2017" OR "2016" OR "2015" OR "2014" OR "2008" OR "2007" OR "2006" OR "2005" OR "2004" OR "2003" OR "2002" OR "2001" OR "2000" OR "2013" OR "2012" OR "2011" OR "2010" OR "2009"))
